# An Assessment of the Paediatric Cardiovascular Risk Profile in San Nicola da Crissa, a Village in the Calabria Region (Southern Italy): A Cross-Sectional Study

**DOI:** 10.3390/jcdd13050207

**Published:** 2026-05-13

**Authors:** Francesco Martino, Angela Sciacqua, Tarcisio Niglio, Francesco Barillà, Eliana Martino, Marco Alfonso Perrone, Pier Paolo Bassareo

**Affiliations:** 1 Department of Internal Medicine, Anaesthesiology, and Cardiovascular Sciences, La Sapienza University of Rome, 00161 Rome, Italy; francesco.martino46@libero.it (F.M.); elianamartino@inwind.it (E.M.); 2 Department of Medical and Surgical Sciences, University Magna Graecia of Catanzaro, 88100 Catanzaro, Italy; sciacqua@unicz.it; 3 Istituto Superiore di Sanità, 00161 Rome, Italy; posta@tarcisioniglio.it; 4 Department of Cardiology, Unicamillus, Saint Camillus International University of Health Sciences, 00131 Rome, Italy; 5 Division of Cardiology and Cardio Lab, Department of Clinical Science and Translational Medicine, University of Rome Tor Vergata, 00133 Rome, Italy; marco.perrone@uniroma2.it; 6 School of Medicine, University College of Dublin, D04ViW8 Dublin, Ireland; 7 Mater Misericordiae University Hospital, D07R2WY Dublin, Ireland; 8 Children’s Health Ireland at Crumlin, D12N512 Dublin, Ireland

**Keywords:** primary prevention, cross-sectional study, intima–media thickness, blood pressure, hypercholesterolaemia, family history

## Abstract

**Background.** Atherosclerosis is a long-standing process that starts in childhood and leads to a number of major adverse cardiovascular events in adulthood. It is therefore crucial that children at potential risk of atherosclerosis-related harmful consequences are identified. Nevertheless, relatively few studies have focused on primary prevention in paediatric patients. **Methods.** Fifty-four children (mean age 9.0 ± 2.8 years) and 72 parents (mean age 44.0 ± 8.2 years) were recruited. Blood pressure (BP) was measured and lipid panel was checked, together with carotid intima–media thickness (IMT) and several indexes of carotid stiffness. **Results.** No statistically significant differences in IMT and indexes of carotid stiffness were detected between children and parents, with the exception of the alpha index (*p* < 0.05). In children, IMT was correlated with the alpha index (*p* = 0.01). Seventeen children (31%) had a pathological IMT. The diastolic BP difference between children with normal and pathological IMT was statistically significant (*p* < 0.05). Parents’ total, LDL and HDL cholesterol, as well as triglyceride levels, differed statistically from those of children with both physiological and pathological IMT: *p* < 0.05 for all differences. Children with hypercholesterolemia had a three-fold higher likelihood of having a pathological IMT than children with normal cholesterol (*p* < 0.01). Among children with pathological IMT, 59 percent had one and 41 percent had two parents who were affected by pathological IMT. **Conclusions.** Carotid stiffness was similar in children and their parents, suggesting early familial influences on vascular properties. Many children had a pathological carotid IMT, highlighting how subclinical atherosclerosis is diffuse even at a young age. IMT in children was associated with cholesterol levels, underscoring the importance of early lipid screening and management. The strong association between pathological IMT in both children and their parents supports the hypothesis of a shared genetic or environmental predisposition to early vascular alterations.

## 1. Introduction

Cardiovascular diseases (CVDs) represent the leading cause of death worldwide [1,2]. A recently published international study demonstrated a reduction in life expectancy in Europe, including Italy, from 2011 onwards. Specifically, life expectancy in Italy has been reduced by about 4 months. Conversely, in the period 1990–2011, life expectancy was on an upward trend, with CVD being the main cause underlying this reduction [3].

In Italy, 230,000 subjects per year die from CVD. Of these, 47,000 deaths are attributed to the lack of regular lipid panel checks. Other contributing factors include the widespread presence of classic cardiovascular risk factors. CVDs represent the last stage of atherosclerosis, a chronic and progressive inflammatory process involving the wall of medium- and large-size arteries which starts in childhood. Atherosclerosis gradually progresses asymptomatically over the years, ultimately triggering the onset of CVD [4,5]. Long-term case–cohort studies have revealed how children with cardiovascular risk factors have a tendency to develop preclinical atherosclerosis and early adverse cardiovascular events [6,7,8].

A key factor in the development of atherosclerosis is represented by high circulating levels of oxidated low-density lipoproteins. These increase oxidative stress and activate monocytes and platelets which, in turn, release pro-inflammatory, pro-atherogenic, pro-oxidant and vasoconstrictive substances [9,10,11].

A recent survey was conducted on 46,309 Italian children and adolescents (mean age 9.7 ± 2.3 years; age range 6–14 years; males 50.48%) to investigate awareness of their total cholesterol levels. However, only a small minority of subjects (4.33%) were aware of this crucial value [12]. Although the majority of apparently healthy adolescents have no atherosclerotic lesions, in some, the clustering of risk factors has been linked to acceleration of atherosclerotic lesions in their coronary arteries [13]. Indeed, primary cardiovascular prevention plays a pivotal role in detecting children and adolescents who are at risk of developing CVD early in life [14,15,16,17,18,19].

The adverse effects of atherosclerosis can be prevented, at least in part, by means of early detection of and actions targeting cardiovascular risk factors [20,21]. Primary cardiovascular prevention should be implemented in childhood by optimising physical activity, body mass index, blood glucose and total cholesterol levels, blood pressure, and sleep, and avoiding the use of tobacco [22,23]. Case–cohort studies have shown that early exposure to cardiovascular risk factors such as family history of myocardial infarction, dyslipidaemia, high blood pressure and smoking are associated with early onset of atherosclerosis and related adverse cardiovascular events [24]. This kind of study may be of use in the early identification of cardiovascular risk factors in childhood. Moreover, other studies have demonstrated a higher prevalence of predisposing factors including diabetes, dyslipidaemia, and metabolic syndrome in the offspring of parents who have experienced an early heart attack [25,26]. Furthermore, unhealthy behaviours (such as smoking, physical inactivity, poor diet and alcohol abuse) are likewise associated with the early onset of CVD, frequently in childhood or adolescence [27]. In a previous study, we observed how the offspring of patients with premature myocardial infarction represented a population at high cardiovascular risk. Indeed, they displayed increased levels of oxidative stress, platelet activation, and elevated levels of nicotinamide adenine dinucleotide phosphate oxidase (NOX-2), isoprostanes, and serum thromboxane [28].

The aim of this cross-sectional study was to identify very young subjects displaying early structural and functional vascular changes known to predict adverse cardiovascular events.

## 2. Materials and Methods

The study was carried out in children and adolescents residing in the municipality of San Nicola da Crissa (Vibo Valentia, Italy) and attending primary and junior high schools.

Lipid panel assessment was performed on venous blood drawn from the antecubital fossa. Blood pressure (BP) was measured by means of an automatic, non-invasive oscillometric device (Dinamap Compact T; Johnson & Johnson Medical Ltd., Newport, UK) at the brachial artery of the dominant arm. BP was measured five times over a 10 min period and the mean of the last three measurements was used. In addition, subjects enrolled in the study underwent an ultrasound scan of the carotid arteries with Doppler sampling to measure intima–media thickness (IMT) together with several indexes of arterial distensibility. The carotid ultrasound scan was performed using an Esaote MyLab ultrasound system (EsaoteTM, Genova, Italy), with patients lying in the supine position and with a slight neck extension in the region of the carotid artery. A probe working at 7.5 MHz and the Q-Elaxto version 13.60 software package (EsaoteTM, Genova, Italy) were used, while quality arterial stiffness (QAS) 13.60 software was used to derive arterial stiffness-related parameters by means of radio-frequency echo-based wall tracking. To perform acquisitions, the probe was placed along a longitudinal axis passing along the distal part of the carotid artery just below the atherosclerotic plaque. The QAS algorithm allowed us to automatically derive real-time measurements of the change in diameter of the vessel between systolic and diastolic phases. The carotid pressure waveform was derived from the brachial pressure and cross-sectional area of the vessels during the cardiac cycle. From the ultrasound images, QAS allowed us to derive different markers of arterial stiffness from the changes in vessel area in relation to local pressure during diastolic and systolic phases: (a) distensibility coefficient (DC), expressed in 1·kPa^−1^ and defined as DC = ∆A/(A·Δp), where ΔA is the change in area during systole, A is the diastolic area of the vessel, and Δp is the local pulse pressure; (b) compliance coefficient, expressed in mm^2^·kPa^−1^ and defined as DC = ΔA/Δp; (c) index of alpha stiffness (α index), representing the elastic coefficient of the vessel calculated as α = A·ln(SAP/DAP)/ΔA, where SAP and DAP are systolic and diastolic pressure, respectively, and ln is the natural logarithm; (d) index of beta stiffness, i.e., the elastic coefficient normalised by vessel diameter, calculated as β = D·ln(SAP/DAP)/ΔD, where D is the diastolic diameter and ΔD is the change in diameter during systole; (e) pulse wave velocity, calculated in m∙s^−1^ as PWV = 1/√(ρ·A − Δp/ΔA), where ρ is the blood density and Δp is local pulse pressure [29]. Carotid ultrasonography was performed using linear probes of 7.5 to 10 MHz. IMT was defined as the distance between the leading edge of the lumen–intima echo and the leading edge of the media–adventitia echo. Measurements were taken bilaterally at the left and right carotid arteries at a site free of plaque 10 to 15 mm proximal to the carotid bulb. The mean of measurements was used for statistical purposes. Values are expressed in millimetres. In the presence of a plaque, defined as a focal lesion >1.2 mm thick, measurements were performed before or after the plaque. All measurements were performed offline by a single experienced examiner who was blind to subjects’ clinical and laboratory findings [30].

Each subject, or their legal representative if the subject was underage, read and signed an informed consent form. The study was formally approved by the Ethics Committee of the Calabria Region (Italy) (PG/108/17.04.2018) and carried out in accordance with the Declaration of Helsinki. Data collection was initiated in September 2021, due to the COVID-19 restrictions in place in Italy at the time. As per Italian law, an Ethics Committee approval remains valid for a period of 10 years.

### Statistical Analysis

Data were collected in a Microsoft Access database and analysed using Epi-Info 7 programmes (CDC and NIH, 2022 Italian version 7.2.5.0). Statistical analysis estimated descriptive statistics, frequencies, and significance of shown differences. Statistical significance “between” and “within” groups was calculated using continuous variables. Analysis of variance (ANOVA) was performed to test the equality of means between pre- and post-treatment for continuous variables, including Bonferroni and Newman–Keuls pairwise mean comparison tests. The Mann–Whitney test was also used by the Statcalc 7.2.5.0 and Analysis 0.5.7 programmes. A *p* level < 0.05 was considered significant.

## 3. Results

Ninety-three children and adolescents (46 females and 47 males; mean age of females 9 ± 2 years; mean age of males 10 ± 3 years), referred to as probands, were initially enrolled in the study. Their parents (n = 86; mothers n = 44, mean age 41 ± 6 years; fathers n = 42, mean age 47 ± 8 years) were also enrolled. Probands included all children attending primary and junior high schools in San Nicola da Crissa, a village in the province of Vibo Valentia in the Calabria region (southern Italy).

Out of the 93 recruited probands, only 54 (28 females and 26 males) underwent carotid artery ultrasound examination. Among the enrolled 86 parents, a total of 72 (37 females and 35 males) agreed to undergo the same scan. These numbers, however, remained statistically representative of the analysed cohort. Indeed, with an expected prevalence of 10% and the absolute precision of the 95% confidence interval, if a sample of 54 is taken from a population of 93 children, the sample size may be reduced to as few as 34 individuals. Therefore, the 54 analysed subjects are more than sufficient and representative of the paediatric school population in San Giovanni di Crissa.

The mean age of the 54 probands was 9.0 ± 2.8 and the mean age of the 72 parents was 44.0 ± 8.2.

The baseline characteristics of the probands and their parents are summarised in Table 1.

Ultrasound and Doppler scan of the carotid arteries revealed the values found in Table 2. The only statistically significant difference lay in the difference in Alpha index (2.53 ± 1.74 vs. 3.88 ± 2.10, *p* < 0.05) between probands and parents. No other differences were statistically significant (*p* = ns).

**Figure 1:**
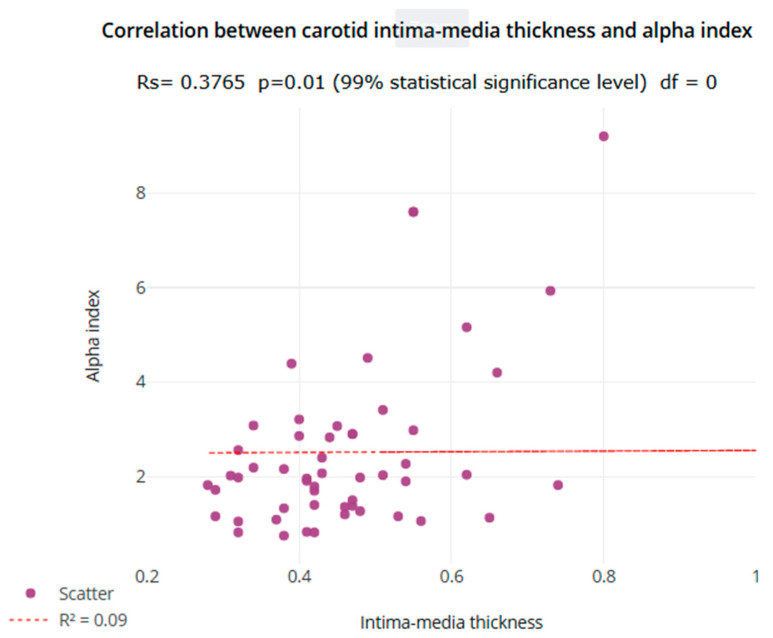
Correlation between IMT and alpha index.

In probands, IMT values were correlated with the alpha index (*p* = 0.01) (see Figure 1).

With regard to IMT measurement, 17 out of 54 probands (31% of the sample—6 females and 11 males) had a pathological value > 0.5 mm.

As for BP values, the difference in diastolic BP between those with IMT > 0.5 mm and those with IMT ≤ 0.5 mm was statistically significant (75 ± 11 vs. 73 ± 10 mmHg, *p* < 0.05). No other differences reported in Table 3 achieved statistical significance (*p* = ns).

Values obtained in the lipid panel assessment by probands and parents are reported in Table 4. Differences in values obtained by parents compared to probands for total cholesterol, LDL cholesterol, HDL cholesterol and triglycerides achieved statistical significance in the presence of both physiological (≤0.50 mm) and pathological IMT (>0.50 mm): *p* < 0.05 for all differences. No statistically significant differences were found between probands with IMT < 0.5 mm and those with IMT > 0.05 mm.

The same statistically significant differences were found after stratifying parents according to the IMT status of their children (see Table 5 and Table 6).

Probands affected by hypercholesterolemia (i.e., total cholesterol ≥ 170 mg/dL) had a three-fold higher likelihood of having a pathological IMT (>0.5 mm) than probands with normal cholesterol (<170 mg/dL). This difference was statistically significant (*p* < 0.01). Among children with pathological IMT > 0.5 mm, 59 percent had one and 41 percent had two parents who were affected by pathological IMT (>1 mm).

## 4. Discussion

By preventing cardiovascular diseases early in life, the long-term healthcare costs associated with treating these conditions can be reduced. CVDs are among the leading causes of death worldwide, producing an immense financial burden on healthcare systems. By preventing CVDs from a young age and thus decreasing the prevalence of these diseases, healthcare costs are reduced in the long term. This was the main reason underlying the decision to conduct a primary prevention cross-sectional study in a small village in southern Italy.

Among all the evaluated elastographic markers of carotid stiffness, it is remarkably surprising that no statistically significant differences were detected between the children recruited in the study and their parents, with the exception of the alpha index. Indeed, carotid distensibility was similar in both groups, regardless of the marked difference in age, implying that a significant portion of arterial elasticity might be determined by hereditary (genetic) factors and shared early life environmental exposures, rather than solely by the ageing process itself [31]. This was despite the significant difference in total, LDL, and HDL cholesterol, and triglyceride levels between parents and children with normal as well as pathological IMT values.

Amongst the markers of carotid stiffness, the only statistically significant difference between the paediatric population enrolled in the study and their parents was obtained for the alpha index, indicating a higher carotid stiffness in the latter. The alpha index is a measure used to assess elasticity of the carotid artery, providing an indicator of arterial stiffness. Higher values of the carotid alpha index typically signify greater stiffness, which is associated with a higher risk of CVD. It is useful in clinical practice to detect early changes in arterial health and assess the risk of cardiovascular events, particularly in patients with conditions such as hypertension or diabetes. The higher the carotid alpha index, the stiffer the artery becomes, potentially indicating early vascular changes [32,33]. A strong relationship between alpha index and carotid IMT was found in the children studied. This point is controversial, particularly as the limited studies performed to date have demonstrated how increased IMT in children was accompanied by higher arterial compliance and lower stiffness [34]. Further research will be needed to clarify the issue.

Another unexpected observation was that about one third of probands had a pathological IMT value (>0.5 mm) [18]. In children, a pathological IMT indicates early or subclinical atherosclerosis and endothelial distress, a key risk factor for future CVD events such as heart attacks and strokes later in life [35,36,37]. In addition, children with pathological carotid IMT have higher diastolic blood pressure values than those with normal IMT. Accordingly, even in children, the close relationship between IMT and blood pressure is confirmed. A large number of cross-sectional studies conducted in young children have suggested that raised blood pressure is closely correlated with the rise in IMT in people with and without hypertension [38,39]. The lack of any difference regarding systolic blood pressure is likely due to the small sample size enrolled in this cohort study.

In addition, children with total cholesterol ≥ 170 mg/dL are three times more likely to have a mean IMT > 0.5 mm than subjects with total cholesterol < 170 mg/dL. This difference is statistically significant (*p* < 0.01). It is indeed an ascertained fact that even in children, higher total cholesterol levels are linked to greater IMT, indicating that the carotid artery wall thickens as cholesterol increases. This occurs as excess cholesterol—particularly LDL cholesterol—promotes plaque buildup within arterial walls, which contributes to atherosclerosis and leads to increased IMT [40].

Finally, pathological IMT in children was linked with pathological IMT values in their parents, since in 59% of probands with pathological IMT > 0.5 mm, one or both (41%) parents were affected by pathological IMT (>1 mm). This is a novel finding, as previously only a modest mother–child IMT concordance had been demonstrated. Nevertheless, in the previous research study, the sample recruited comprised a large proportion of mothers, limiting generalisability of concordance findings for fathers [41].

The present study was hampered by a few limitations, namely: a small sample size, which may not be representative of the general paediatric population in southern Italy; the possibility that participants may not accurately remember past exposures which may influence the outcome, potentially creating false associations; the possibility that a cross-sectional study may not be the best design with which to study subclinical atherosclerosis in the paediatric population due to the presumed uncommon nature of the condition and consequent difficulty in recruiting a sufficiently high number of cases; due to the cross-sectional study design, no cause-and-effect relationship could be established with any degree of certainty. However, the study findings seem to be promising despite the lack of a follow-up period resulting in a lack of data relating to the potential development of CVD.

## 5. Conclusions

In this paediatric cross-sectional study, values obtained for carotid stiffness were comparable between children and their parents, suggesting likely early familial influences on vascular properties. Notably, approximately one third of children demonstrated a pathological carotid IMT, highlighting how subclinical atherosclerotic changes are diffuse and may be detected even at a young age. IMT in children was significantly associated with cholesterol levels, underscoring the importance of early lipid screening and management. Additionally, the strong association between pathological IMT in children and their parents supports the hypothesis of a shared genetic or environmental predisposition to early vascular alterations. Together, these findings emphasise the need for early familial cardiovascular risk assessment and reinforce the importance of implementing preventive strategies in childhood.

## Figures and Tables

**Table 1:** Main features of probands and their parents.

Probands Parents		
Age (years)	9.0 ± 2.8	44.0 ± 8.2
Systolic BP (mmHg)	101.5 ± 16	112 ± 13
Diastolic BP (mmHg)	74.5 ± 10.5	77 ± 7
Total cholesterol (mg/dL)	146.5 ± 31.5	206 ± 29
LDL cholesterol (mg/dL)	86.5 ± 24.5	125 ± 28
HDL cholesterol (mg/dL)	45.5 ± 8.5	46 ± 14
Triglycerides (mg/dL)	76 ± 32.5	171 ± 116

**Table 2:** Differences between probands and their parents with regard to IMT, alpha index, beta index, distensibility coefficient, compliance coefficient, and pulse wave velocity.

Probands Parents		
IMT (mm)	0.46 ± 0.12	0.61 ± 0.12
Alpha index	2.53 ± 1.74	3.88 ± 2.10 *
Beta index	5.15 ± 3.43	7.77 ± 4.09
Distensibility coefficient	390 ± 242	248 ± 143
Compliance coefficient	1.46 ± 0.77	0.98 ± 0.49
Pulse wave velocity	4.38 ± 1.87	5.68 ± 1.44

* *p* < 0.05. No other differences were statistically significant.

**Table 3:** Differences between probands with IMT > 0.5 mm and those with IMT < 0.5 mm with regard to systolic blood pressure, diastolic blood pressure, and heart rate.

	Systolic Blood Pressure (mmHg)	Diastolic Blood Pressure (mmHg)	Heart Rate (bpm)
**Probands (IMT > 0.50 mm)**	103 ± 18	75 ± 11 *	86 ± 12
**Probands (IMT ≤ 0.50 mm)**	100 ± 14	73 ± 10 *	89 ± 11
**Parents**	112 ± 13	77 ± 7	73 ± 10

* *p* < 0.05. No other differences were statistically significant.

**Table 4:** Differences between probands with IMT > 0.5 mm and those with IMT < 0.5 mm with regard to total cholesterol, LDL cholesterol, HDL cholesterol, and triglycerides.

	Total Cholesterol (mg/dL)	LDL Cholesterol (mg/dL)	HDL Cholesterol (mg/dL)	Triglycerides (mg/dL)
**Probands (IMT > 0.50 mm)**	145 ± 24	84 ± 17	48 ± 10	68 ± 27
**Probands (IMT ≤ 0.50 mm)**	148 ± 39	89 ± 32	43 ± 7	84 ± 38
**Parents**	206 ± 29 *	125 ± 28 *	46 ± 14 *	171 ± 116 *

* *p* < 0.05. No other differences were statistically significant.

**Table 5:** Differences between probands with IMT > 0.5 mm and those with IMT < 0.5 mm with regard to systolic blood pressure, diastolic blood pressure, and heart rate, after stratifying parents according to the IMT status of their children.

	Systolic Blood Pressure (mmHg)	Diastolic Blood Pressure (mmHg)	Heart Rate (bpm)
**Probands (IMT > 0.5 mm)**	103 ± 18	75 ± 11	86 ± 12
**Parents with children with IMT > 0.5 mm**	125 ± 13	79 ± 9	75 ± 10
**Probands (IMT < 0.5 mm)**	100 ± 14	69 ± 11	89 ± 13
**Parents with children with IMT < 0.5 mm**	114 ± 15	75 ± 10	75 ± 6

No differences were statistically significant (*p* = ns).

**Table 6:** Differences between probands with IMT > 0.5 mm and those with IMT < 0.5 mm with regard to total cholesterol, LDL, cholesterol, HDL cholesterol, and triglycerides, after stratifying parents according to the IMT status of their children.

	Total Cholesterol (mg/dL)	LDL Cholesterol (mg/dL)	HDL Cholesterol (mg/dL)	Triglycerides (mg/dL)
**Probands (IMT > 0.5 mm)**	145 ± 24	84 ± 17	48 ± 10	68 ± 27
**Parents with children with IMT > 0.5 mm**	201 ± 29 *	123 ± 24 *	48 ± 14 *	149 ± 91 *
**Probands (IMT < 0.5 mm)**	140 ± 21	78 ± 16	47 ± 8	66 ± 23
**Parents with children with IMT < 0.5 mm**	181 ± 41 *	111 ± 42 *	47 ± 10 *	112 ± 80 *

* *p* < 0.05. No other differences were statistically significant.

## Data Availability

The data supporting reported results are available by contacting Tarcisio Niglio on reasonable request.
